# A computational grounded theory based analysis of research on China’s old-age social welfare system

**DOI:** 10.3389/fpubh.2025.1556302

**Published:** 2025-04-28

**Authors:** Agudamu, Yingying Li, Na Mi, Xinyue Pan

**Affiliations:** ^1^Graduate School of Social Welfare, Sungkyunkwan University, Seoul, Republic of Korea; ^2^College of Physical Education, Hunan Normal University, Changsha, China; ^3^College of Physical Education, China University of Mining and Technology, Xuzhou, China

**Keywords:** natural language processing, latent Dirichlet allocation, social insurance, social security, topic modeling

## Abstract

**Purpose:**

By the end of 2024, 22% of the Chinese population was aged 60 and above, making old-age social welfare a critical challenge. Despite abundant literature, a gap remains between research and policy. This study applies Nelson’s computational grounded theory to systematically analyze China’s old-age social welfare research and propose targeted policy priorities.

**Methods:**

We searched Chinese literature (2014–2024) from the Wanfang, CNKI, and CQVIP databases. After preprocessing the abstracts, we applied topic modeling using the latent Dirichlet allocation, guided by human analysts. Optimal topics were determined using perplexity and coherence metrics. Researchers then linked each topic to sociologically meaningful concepts to derive abstract policy conclusions.

**Results:**

A total of 413 articles met eligibility criteria. Seven topics emerged: (1) the theoretical significance of social welfare policy; (2) enhancing rural old-age care; (3) providing care for special groups; (4) promoting a home-community care model; (5) optimizing precision care through collaborative mechanisms; (6) developing community culture; and (7) establishing supply-driven care services. Notably, topics two and seven dominated the literature.

**Conclusion:**

Based on these themes, we propose policy priorities to enhance comprehensive social welfare programs. China’s big government model—a top-level design involving diverse stakeholders—may serve as an effective framework for addressing a global aging society marked by rising non-communicable diseases and AI-driven economic growth. Moreover, our computer-assisted approach offers a valuable method for information scientists, aiding policymakers in navigating extensive digital data for more cost-effective and timely decision-making.

## 1 Background

Advances in healthcare, nutrition, and socioeconomic conditions have significantly increased human lifespans, bringing unprecedented population aging to the forefront (1). This trend is set to become one of the 21st century’s most significant social phenomena, posing multifaceted challenges for individuals, families, societies, and global governing systems. According to the United Nations’ World Social Report 2023 (2), the global population aged 65 and older reached 761 million in 2021 and is projected to rise to 1.6 billion by 2050. In recent years, East Asian countries such as China, Japan, and South Korea—characterized by low fertility rates—have experienced accelerated aging, thereby placing substantial pressure on their social welfare systems (3, 4). In aging societies, welfare policies, particularly those governing healthcare and pensions, critically influence individual well-being and societal stability. As the world’s second most populous nation, China faces a rapidly aging population. By the end of 2024, approximately 22.0% of its population is aged 60 and above, and 15.6% is aged 65 and above (5). By 2050, individuals aged 55 to 64 will comprise 26.7% of the workforce (6). Without significant reforms, declining labor force participation could threaten or even deplete the pension insurance fund (7). Additionally, an aging population increases basic medical insurance expenditures (8), thereby intensifying fiscal pressure on the social welfare system (9). This demographic shift has heightened public concern over social welfare. In addition, China’s rapid economic growth over the past two decades has improved social welfare benefits. However, escalating trade tensions with developed economies, such as the United States, may jeopardize this growth engine and, consequently, the government’s capacity to finance social welfare initiatives. Ironically, the previous export-driven development model could hinder progress toward a broader and more robust social welfare system. Thus, both domestic demographic shifts and a de-globalization trend present unprecedented challenges to designing and implementing effective policies for an aging society.

China has introduced policies to address population aging and enhance old-age welfare, as comprehensively reviewed by Feng and colleagues (10). However, the socialization of old-age welfare began relatively late, with limited private capital support. Consequently, the long-term care insurance system has been piloted only in select cities (4), providing services for older adults requiring extended care. The Law of the People’s Republic of China on the Protection of Rights and Interests of the Elderly was enacted in 1996 and revised in 2015 to define the basic rights of older adults, including access to care, medical services, pension insurance, and cultural engagement. Additionally, the government introduced the “9,073” model, wherein 90% of older adults rely on family and community-based services, 7% on government-run eldercare institutions, and 3% on privately operated facilities. Despite these initiatives, China’s pension insurance system remains limited in scope and unable to fully meet the challenges of an aging population. Key issues include significant funding shortages for welfare programs, an insufficient number of welfare institutions, underdeveloped market-based old-age care options, and a marked mismatch between the demand for and supply of services (10). As a result, the overall development of the old-age care system lags behind the growing needs of the aging population.

Therefore, this study systematically reviews research on China’s old-age social welfare system to propose optimization strategies. To overcome the vast volume of data and mitigate human bias inherent in traditional systematic review, this study will employ natural language processing techniques to semi-automatically sort and categorize research outputs, thereby generating more objective and holistic policy recommendations. The expected findings aim to enhance fairness and sustainability, ensuring that the aging population enjoys a higher quality of life in later years. This research may also offer a “China governance model” for other developing countries facing future population aging and significant economic transitions in the age of artificial intelligence.

## 2 Theoretical framework

### 2.1 China’s social welfare system

Several leading academic journals have mistakenly used “social security” and “social insurance” interchangeably (11–13). To clarify, it is important to outline China’s social welfare system. This distinction is crucial for understanding the policies and structures that address the needs of older adults and other vulnerable groups.

Social insurance is a voluntary program available to both urban and rural residents. Participants contribute monthly premiums for a legally defined period and, in return, become eligible for benefits. Essentially, the government manages a fund into which contributions are made, and from which payments are later distributed. In contrast, social security refers to the government’s allocation of funds not only to those enrolled in the social insurance system but also to uninsured citizens who qualify for financial assistance on humanitarian grounds. Social security expenditures are categorized into four main areas: social insurance, social assistance, social welfare, and special social care. A portion of these funds covers the annual deficit in basic pension insurance, while additional funds support social assistance for individuals with disabilities, disaster victims, social welfare programs, and special care for military personnel and families of martyrs. In essence, government revenue finances these social security expenditures, collectively forming China’s social welfare system. This study provides an overview of research on China’s social welfare system.

### 2.2 State of knowledge

A wealth of literature on China’s social welfare system can be divided into theoretical qualitative research and evidence-based quantitative studies. Here, we highlight a few empirical studies pertinent to our policy recommendations:

Using difference-in-differences estimation, Liu and colleagues examined the relationship between social (pension) insurance contributions and corporate financing decisions (14). Their analysis revealed that higher social insurance co-contributions lead firms, especially those with greater labor market frictions, higher labor intensity, poorer financial health, tighter financial constraints, or located in low-income areas with less developed financial systems, to issue less debt. Similarly, Yu and colleagues found that increased mandated social insurance co-contributions prompt some firms to reduce their workforce (15). Conversely, other research indicates that offering higher tax incentives to firms can boost social insurance co-contributions and, consequently, improve employee pension benefits (16). These findings imply that social insurance policy significantly influences both employees’ retirement benefits and corporate growth. Thus, in revising social insurance policy, the Chinese government must balance the goal of enhancing future retirees’ benefits with the imperative of sustaining current economic growth.

China’s two-track medical insurance system—comprising employment-based insurance and urban–rural resident basic medical insurance—and regional economic disparities are widely recognized contributors to health inequality. Zhang and colleagues demonstrated that although the national concentration index for medical insurance benefits decreased between 2014 and 2020 (a higher index indicates greater inequality), poorer cohorts continued to experience more illness while receiving a smaller share of benefits than wealthier groups (17). Similarly, physical multimorbidity is more prevalent in poorer regions and is significantly associated with an increased likelihood of catastrophic medical expenditure (18). The authors suggested that reforms should focus on reducing out-of-pocket expenses for patients with multimorbidity to enhance financial risk protection. In addition to policy design flaws, limited medical resources further restrict older adults’ access to care. Li and colleagues found that older adults reported the highest levels of unmet hospitalization needs across all demographic groups and recommended developing social programs and home-based care to improve healthcare accessibility (19). These findings underscore that addressing the funding gap in the medical insurance system will require legislative reforms to reduce benefit disparities between urban and rural residents, as well as among self-employed groups. Furthermore, increased fiscal expenditure will be needed to support new medical infrastructure and expanded social security contributions in the face of a growing aging population.

The government could address funding gaps in the pension and basic medical insurance systems by utilizing social security funds to cover deficits. Although a funding gap already exists for pension insurance, medical insurance remains unaffected at present (20, 21). Addressing these issues requires policy reforms, including the delayed retirement age enacted in 2024 and the relaxation of outdated childbirth policies (22). Moreover, a flexible yet targeted monetary policy is crucial. Such a policy would grant the government greater fiscal flexibility to increase social security expenditures—a measure supported by several national-level policy proposals (9, 23, 24).

The literature is extensive, yet three key issues emerge. First, researchers are often influenced by confirmation bias. For example, our natural science training predisposes us to favor empirical evidence, resulting in a predominance of quantitative studies in this review. Nonetheless, qualitative methods are invaluable and addressing this bias is essential for ensuring objective research summaries. Second, natural scientists typically rely on systematic frameworks—such as umbrella reviews, meta-analyses, and network meta-analyses—to integrate qualitative and quantitative evidence. In contrast, the social sciences lack comparable guidelines, rendering holistic reviews highly dependent on specific schools of thought. The third issue will be addressed in the following section.

### 2.3 From research to policy

The goal of social science research is to generate policy recommendations that shape societal development. However, a persistent gap between research and public policy challenges the translation of findings into actionable solutions. The ideal scenario—where experts generate evidence that policymakers seamlessly incorporate into decision-making—often proves unattainable (25, 26). Nutley (27) examined the policy dilemmas within the UK’s public governance system and identified two critical issues. First, the social science literature often consists of small, *ad hoc* studies with diverse methodologies and varying levels of rigor. This variability limits the development of a robust knowledge base from which policymakers can reliably draw. Second, the fragmented nature of the evidence base makes it difficult to synthesize findings and derive actionable conclusions. The absence of a universally accepted framework for systematic reviews in social science further exacerbates this issue. As a result, despite considerable government funding, much academic research fails to influence policy reform as anticipated.

The inefficient translation of research into policy has undesirable effects. Only a small fraction of research gains visibility in policy networks, typically through advocacy (27). Over time, this selective exposure narrows the range of perspectives influencing policy, which undermines both academic progress and effective governance. Although initiatives—such as institutional mechanisms linking researchers to policy networks (28) and innovative dissemination platforms (29)—have been proposed, an Occam’s razor approach may be most effective: Developing an unbiased, straightforward synthesis method to consolidate and disseminate evidence could significantly improve the research-to-policy pipeline.

### 2.4 Computational grounded theory

In the big data era, social science research has access to exponentially expanding data resources. Traditional manual reviews are labor-intensive (27) and often introduce subjectivity, undermining reproducibility. Nelson’s computational grounded theory (CGT) (30) offers a promising framework to analyze large volumes of unstructured data by uncovering hidden semantic structures and fostering new ideas, concepts, and hypotheses. CGT combines human interpretative skills with computational rigor, preserving the holistic insights of human analysts while benefiting from the formal reliability and reproducibility of computational tools.

The CGT process typically involves three steps. Initially, in pattern detection, computational algorithms decontextualize and simplify textual data, revealing patterns not immediately apparent to human readers. This allows researchers to analyze extensive datasets without manually reviewing the entire corpus, reducing complex text to clusters of interpretable terms that lay the groundwork for further analysis. Next, in the pattern refinement stage, human analysts perform a qualitative exploration of the data, deeply reading and contextualizing the patterns identified computationally. Finally, an optional pattern confirmation step employs additional techniques to validate these inductively derived patterns, ensuring their robustness and reliability. In summary, CGT bridges qualitative and quantitative methodologies, offering social scientists a transparent and reproducible framework for generating data-driven concepts.

Nelson’s CGT is relatively new, with only one study by Polish researchers applying a similar method in social welfare research (31). Accordingly, this study aims to employ CGT to extract key topics from Chinese scientific literature on the old-age social welfare system, uncover hidden thematic structures, and classify and cluster textual content. By utilizing this computational approach, we seek to provide an in-depth synthesis of existing research and propose optimization strategies for developing a comprehensive old-age social welfare system in China.

## 3 Methods

### 3.1 Data preparation

China’s social welfare system has undergone significant reforms, as reviewed by Zheng (32). In 2014, the consolidation of rural residents’ pension insurance and urban non-employed residents’ pension insurance into the urban–rural residents’ pension insurance system marked the foundational development of a social welfare system with Chinese characteristics. Accordingly, this study reviews literature published between 2014 and 2024.

An electronic search was conducted in the Wanfang, CNKI, and CQVIP databases using the Boolean expression (“social security” OR “social insurance” OR “social welfare”) AND “old age.” In natural sciences, systematic literature reviews typically assess each record’s quality before further analysis. However, such quality control is not standard in social sciences. To efficiently filter out low-quality evidence, we applied an automatic filter during the search. Specifically, we considered only documents listed in the Chinese Social Sciences Citation Index, the authoritative source in China’s scientific library system, for further eligibility inspection. Subsequently, irrelevant articles were excluded during title and abstract screening using the following criteria: articles discussing foreign experiences; those focusing on the broad financial impact of old-age policies; theoretical articles addressing law and regulation reforms in old-age policy; and econometric studies on social welfare system reforms for all populations. This procedure was independently carried out by two researchers (A and YL), with discrepancies resolved through discussion.

### 3.2 Modified CGT procedure

**Figure 1:**
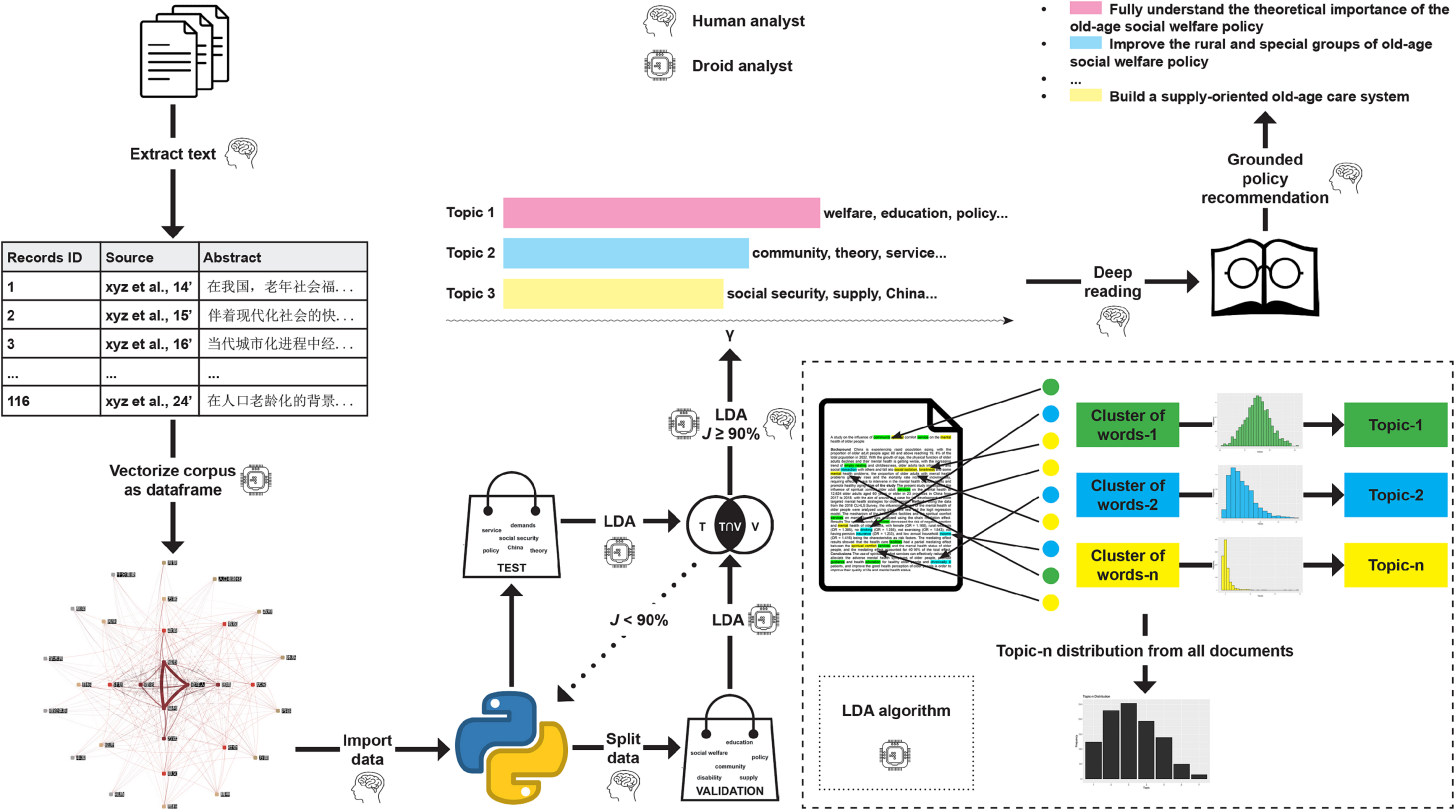
Flow chart of the research method.

In the previous section, we introduced Nelson’s CGT, which involves three steps: pattern detection, pattern refinement, and pattern confirmation (30). Nelson also noted that “different projects may employ these steps in different orders depending on the nature of the data and the research question.” In essence, CGT enables researchers to navigate extensive literature through a computer-assisted, quantitative procedure for identifying topics in a reproducible manner. This approach, grounded in existing evidence, ultimately facilitates the generation of comprehensive policy recommendations. In this study, the term “pattern” refers to the topics identified within the old-age social welfare literature, while “confirmatory” pertains to the reliability of these identified terms and the resulting topics. Based on this framework, we designed a customized CGT procedure, as illustrated in Figure 1.

The pre-processing of the corpus involved extracting, cleaning, and vectorizing data from text into a structured dataframe. Initially, the abstracts from the included literature were converted into digitized text. These texts were then transformed into computer-readable “bag-of-words” to reduce dimensionality. Given the complexity of the Chinese language (33), we incorporated an additional step—lexical selection. This process involves choosing words that effectively convey the intended meaning, align with the discourse’s tone, and remain contextually relevant. In our study, we used Jieba, a Python-based Chinese text segmentation library, to process the texts. Additionally, we filtered out specific stop words—“older adult,” “aging,” “welfare,” “geriatric,” “impact,” “research,” and “analysis”—to avoid interference with subsequent algorithmic text analysis.

Despite the introduction of CGT, open-source algorithms for topic modeling within its framework are rapidly emerging. Among these, latent Dirichlet allocation (LDA) remains the most widely used algorithm in sociology, despite ongoing criticisms (34). LDA employs probability distributions from the Dirichlet family, governed by two hyperparameters—α (document-topic density) and β (word-topic density)—which influence the shape and specificity of the word-topic and topic-document distributions. The fundamental assumption of LDA is that documents are generated from a mixture of topics, with each topic represented as a probability distribution over words. In theory, this method effectively reveals latent topic structures within a document collection, thereby enhancing classification and clustering. In this study, topic modeling was conducted using the LDA-based Gensim library (version 4.3.0) in Python.

Since LDA aims to determine the optimal number of topics, and the terms identified by LDA directly shape the final topics, we implemented a customized pattern confirmation step. Specifically, we randomly selected 70% of the included literature to construct a validation bag-of-words, while the test bag-of-words comprised the full corpus. We compared the similarity of terms between these two sets using default LDA settings. The underlying hypothesis was that if the algorithm is stable and thematic saturation is approaching (35), the similarity between the two bag-of-words should be sufficiently high. To quantify the term similarity, we employed the Jaccard similarity coefficient (36), a metric widely used in various fields including the evaluation of cohesive subgroup density (37) and strategic management (38). The Jaccard coefficient, defined in Equation 1, ranges from zero (no shared terms) to one (identical bag-of-words). Given its novelty in the context of CGT, we set conventional thresholds: a coefficient of 95% indicates “good similarity” and 90% indicates “acceptable similarity.” If the Jaccard similarity fell below 90%, we adjusted LDA hyperparameters, burn-in rate, or iterations, or acknowledged that the literature on China’s old-age social welfare system might not yet have reached thematic saturation.


(1) $$J\left(\bar{\mathrm{Test}},\bar{\mathrm{Validation}}\right)=\frac{\left|S_{\mathrm{Test}}\cap S_{\mathrm{Validation}}\right|}{\left|S_{\mathrm{Test}}\cup S_{\mathrm{Validation}}\right|}$$

A crucial step in CGT is determining the optimal number of topics. Topic modeling blends computer-generated output with human judgment to yield interpretable topics (39). The most commonly used evaluation metric is perplexity on a held-out dataset relative to the trained model. Perplexity measures the probability of unseen data given the model; the underlying idea is that if most words in a document align with the top-ranked words from the topic probability distributions generated by LDA, the model’s topic predictions are more accurate. Thus, lower perplexity indicates better predictive performance. The calculation of perplexity is presented in Equation 2.


(2) $$\mathrm{Perplexity}=\exp\left(\overset{N}{\underset{i=1}{\sum }}-\log p\left(\mathrm{xi}\right)\right)$$

Where, *p*(*x*_i_) is the predicted probability of the model for *i* observations *x*_i_, *N* is the total number of observations in the dataset.

Meanwhile, Chang et al. observed a negative correlation between lower perplexity in LDA models and human judgment (40). This finding underscores the need for additional measures to determine the optimal number of topics for semantic interpretability. Hence, we incorporated the coherence metric into the selection process (41). The coherence metric evaluates a topic by measuring the semantic similarity among its highest-scoring words; a higher likelihood of these words appearing together indicates a better classification effect. Ultimately, the number of topics was determined through evaluation by human analysts, who assessed both the semantic interpretability of the topics and the objective results from statistical inference.

Subsequently, two researchers (A and YL) refined the patterns by reviewing the full texts—specifically, the conclusions and practical implications sections—of the included literature. Guided by computational topic clustering, researchers efficiently extracted the main conclusions from each document and categorized them by topic. These categorizations then served as the basis for developing grounded policy recommendations.

## 4 Results

**Figure 2:**
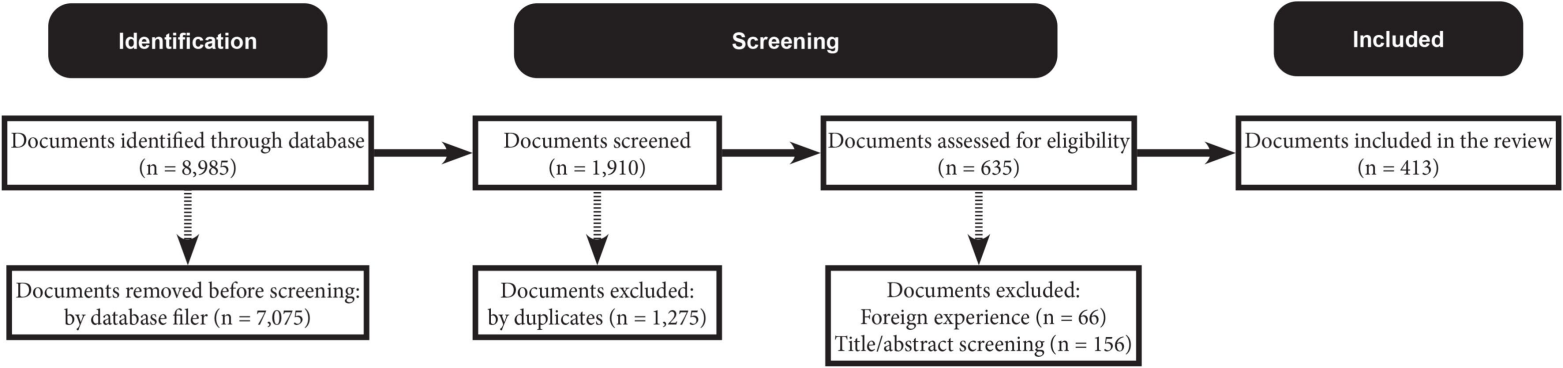
Flow diagram of literature search.

Figure 2 summarizes the search process, resulting in a total of 413 documents included for analysis. A complete list of the included documents is available on Figshare (DOI: 10.6084/m9.figshare.28029059.v4).

Following the preprocessing of lexical data, we compared the similarity between the test and validation datasets using the same algorithm. The Jaccard similarity coefficient was calculated at 95.6%, indicating a substantial overlap between the terms in both datasets. This result suggests that the algorithm demonstrated stability within this dataset, and that including additional documents in the topic modeling is unlikely to yield significantly different outcomes.

**Figure 3:**
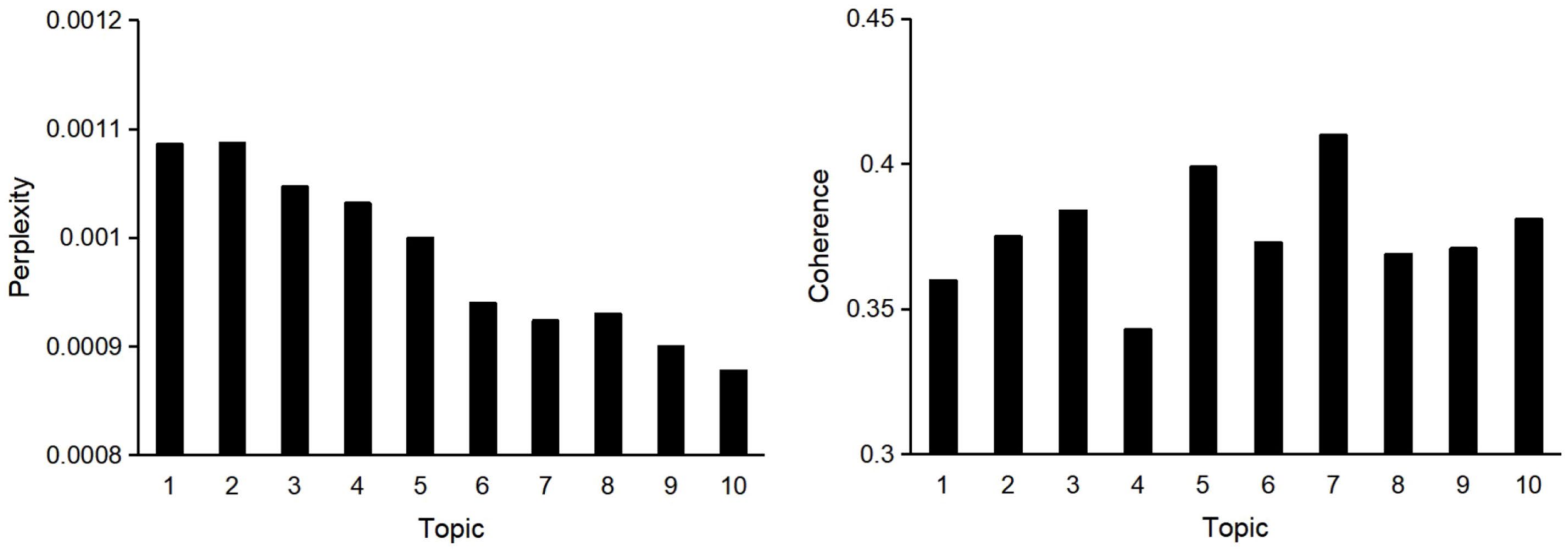
Topic modeling selection criteria.

Figure 3 illustrates the computational criteria used for topic selection. Based on the perplexity metric, models with six to ten topics demonstrated reasonable predictive performance. When combined with the coherence score, the seven-topic model showed an approximately 13.8% improvement over the baseline and outperformed the other models. Additionally, one researcher (A) with domain expertise confirmed that the seven-topic model comprehensively encompassed content areas such as politics, economics, culture, and old-age care services. Therefore, the seven-topic model was deemed optimal for classifying the included documents.

Using the top 10 weighted terms, researchers interpreted the topics as presented in Table 1. For example, topic one focuses on government policies regarding social welfare for old-age care. The analysis indicates that the Chinese government recognizes the importance of supporting the older population and has undertaken proactive initiatives in this area. Researchers in this topic typically examine the implementation and outcomes of the social security system, while also proposing solutions to address its challenges (42, 43).

**Table 1:** Summary of the seven-topic model.

Topic	Topic connotation	Corresponding terms^*^
1	Offer robust theoretical support to enhance old-age care systems and ensure life security for aging populations.	Old-age care, society, security, government, development, life, demand, supply, theory, provide
2	Strengthen policy support to improve social welfare systems for rural old-age care in China, ensuring equitable access to resources and services for aging populations.	Society, policy support, demand, development, supply, security, rural, china, social welfare, service
3	Develop a comprehensive system to address the old-age care needs of people with disabilities, ensuring accessibility and appropriate support services.	Demand, society, satisfy, system, people with disabilities, security, time, special population, education, old-age care
4	Promote the economic development of old-age care through implementation in communities and families, ensuring sustainable support and resources.	Old-age care, support, policy, society, community, family, development, economy, rural, supply
5	Old-age care services and policies designed to meet the diverse care needs of older adults, ensuring comprehensive support and well-being.	Welfare policy, supply, issues, development, social support, service, old-age care, care, propose, needs
6	A community-based cultural system to support social welfare security for the older adults, promoting inclusivity and well-being.	Society, policy, social welfare, older adults, security, system, tradition, culture, community, china
7	Supply-driven old-age care service policies and their impact on social development, focusing on enhancing resources and support for aging populations.	Old-age care, development, society, supply, policy, optimization, issue, family, establish, provide

* The terms are weighted and presented in descending order of importance.

**Figure 4:**
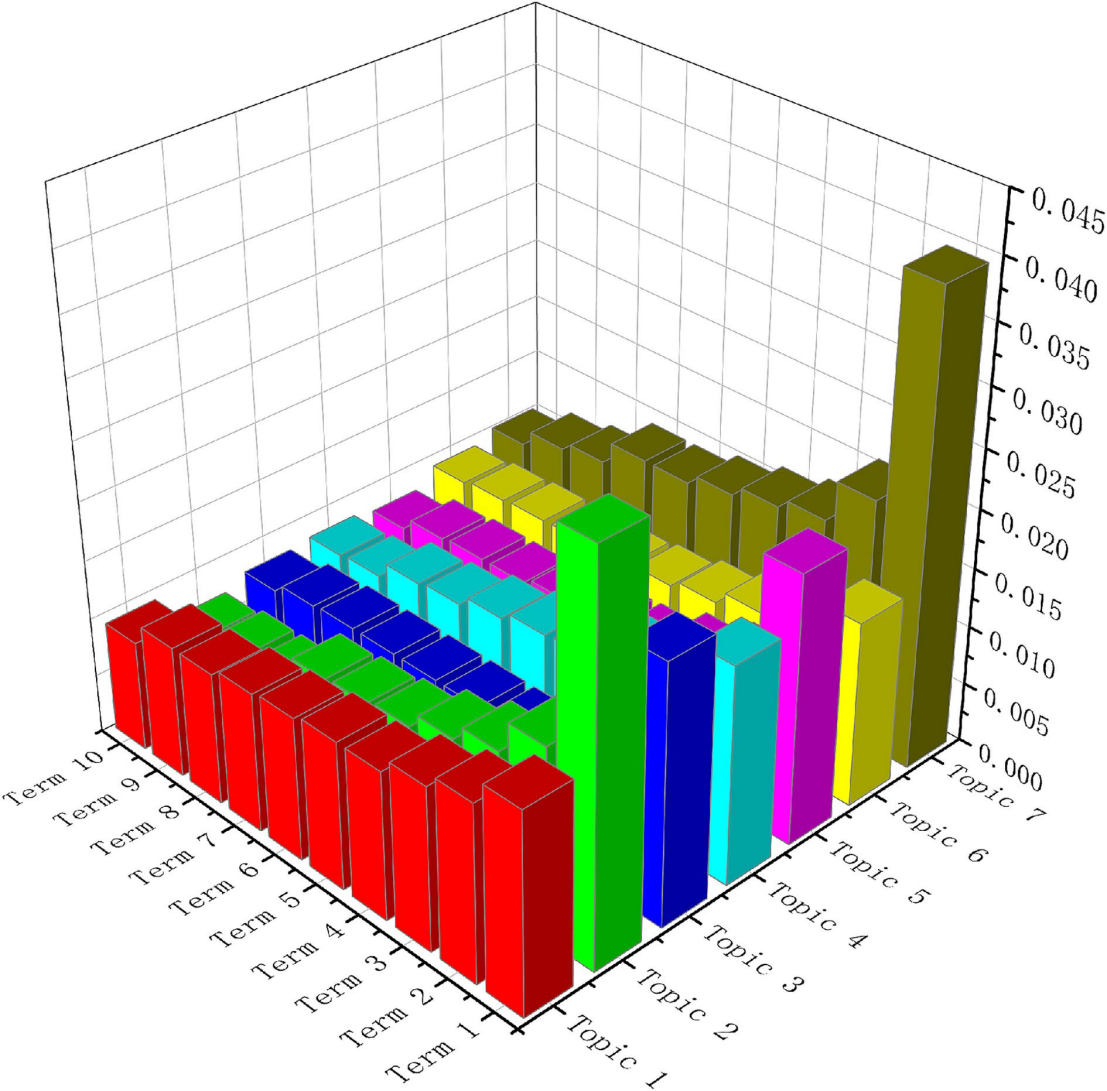
Expected topic proportion along with top 10 terms.

The inter-topic proportions further reveal research trends within the field. As shown in Figure 4, topics two and seven are disproportionately represented in the literature. For example, topic two examines China’s rural old-age care policies, emphasizing policy guarantees for social welfare and a supply-driven approach to service provision. This focus reflects the government’s prioritization of rural old-age care as a critical component of the broader social welfare system. Through targeted policy development and implementation, China aims to establish a more equitable and sustainable social security system, thereby ensuring reliable services and support for its rural older population.

## 5 Discussion

This review employed CGT to analyze topics from 413 Chinese research works on the social welfare system. During pattern refinement, grounded clustering and subjective analysis identified seven key topics: theoretical foundations for old-age care; care for rural or impoverished older adults; care for the disabled; the economics of community-and family-based old-age care; the service needs of older adults; the sociocultural service system for old-age welfare protection; and supply-driven old-age care policies. Accordingly, we propose six optimization recommendations. Notably, to bridge the gap between research and policy (27), we adopted a formal writing style favored by policymakers while maintaining academic rigor.

### 5.1 Recognizing the significance of old-age welfare policies in addressing China’s unique demographic and social needs

Theoretical support provides the foundation for policy formulation and implementation in old-age welfare, thereby promoting social enterprises’ commitment to protecting older adults and enhancing overall societal well-being (42, 44). The 20th National Congress (45) set forth a strategic deployment that targets basic socialist modernization by 2025 and aims to build a prosperous, democratic, civilized, and harmonious socialist modern nation by 2035. This underscores the urgency of developing a social welfare system with Chinese characteristics. China’s social welfare system is primarily guided by centrally planned economic policies, with social policies serving as a supplement. This transformation is especially evident in old-age care, where government initiatives—such as establishing universal pension and medical insurance systems, encouraging a rebound in fertility rates (46), gradually raising the retirement age (47), and promoting smart old-age care (48)—highlight the system’s critical role in addressing population aging.

Old-age welfare protection remains a challenging public issue. It is essential to ensure fairness in the pension system (49) and to adopt a comprehensive approach that includes pension insurance reform, the development of the old-age care industry, fiscal subsidy mechanisms, and robust legal rights systems (50). Such an approach will enable the system to adapt to the evolving dynamics of an aging population and emerging geopolitical challenges, avoiding policy rigidity and maintaining the flexibility needed to respond to new issues.

### 5.2 Enhancing social welfare policies for rural and special groups in old-age welfare programs

The government has increasingly prioritized the provision of reliable services and support to rural older populations. The 20th National Congress emphasized comprehensive rural revitalization, identifying rural old-age care as both a critical task and a necessary response to the challenges posed by rural aging. Rural areas currently face significant issues, including incomplete old-age care facilities (51), the migration of younger populations to urban centers (52), pronounced aging trends (53), and the empty-home phenomenon (54), which undermines traditional family caregiving roles. These challenges underscore the urgent need for a strengthened social welfare system tailored to rural older populations (55). Farmers, operating within market economies, can no longer rely solely on traditional land-based security systems to mitigate risks. Despite government initiatives to provide protective measures, these efforts remain insufficient, leaving farmers vulnerable to major illnesses or natural disasters, often resulting in back to poverty (56). This situation highlights the pressing need for further refinement of the social welfare system to address the specific needs of rural older populations.

In recent years, China has introduced a range of policies to address the growing challenges of rural old-age care amid a rapidly aging population. These initiatives focus on both economic and infrastructure support, as well as the development of robust social welfare systems. For example, the abolition of agricultural taxes, increased agricultural subsidies, and enhanced rural infrastructure have been prioritized to boost farmers’ incomes and improve living standards, thereby elevating the quality of life for rural older populations (57). Concurrently, the government has implemented key programs such as the rural minimum living security system, the new rural social pension insurance, and the rural cooperative medical system to provide essential living and healthcare guarantees for rural older adults (58, 59).

To counteract the weakening of traditional family care and the limitations of market-driven services, the China National Committee on Aging has advocated for rural mutual assistance models. These models are characterized by low-cost, nonprofit operations that encourage broad participation, enhancing the accessibility and effectiveness of old-age care services. Despite these commendable efforts, significant challenges persist in rural old-age care, including ongoing economic pressures on rural families, increased burdens on family caregivers, and a diminishing cultural foundation for traditional care practices (60, 61). Addressing these issues will require further measures, particularly in the area of fiscal support. Expanding fiscal investments to strengthen the rural social security system, allocating additional resources to rural medical institutions, increasing salaries for rural healthcare workers, and incentivizing the redistribution of high-quality healthcare resources to underserved areas are all necessary steps (62). By promoting these measures, China can work toward ensuring that rural residents have access to secure, dignified, and sustainable old-age care services, aligning with the broader goals of equitable and inclusive development.

China’s approach to meeting the old-age care needs of persons with disabilities is primarily embedded within its social security system. Under the “Measures for the Administration of Subsidy Funds of the Central Treasury for the Basic Living Assistance of the Impoverished People,” older adults with disabilities qualify for support. Despite these measures, older adults in extreme poverty continue to face significant challenges, including a low quality of life and difficulties in safeguarding their rights (63–65). Consequently, further action is needed to enhance benefits and protections for this vulnerable group.

Looking ahead, a synergistic effort among government, market, and society is essential (66). The government should implement more preferential policies for the old-age care of persons with disabilities, such as tax reductions and subsidies, to incentivize broader social participation in these services (66, 67). In addition to direct government funding, alternative financial resources could be mobilized through social donations and commercial insurance mechanisms. Prioritizing comprehensive rehabilitation and therapy services—including both physical treatment and psychological counseling—is crucial to improving their functional abilities and overall well-being (68). Recognizing that older adults in extreme poverty may continue to face hardships in the short term, it is imperative to refine related policies and promote the high-quality development of the old-age care service system. Such advancements will not only enhance the quality of life for persons with disabilities but also contribute to national harmony, stability, and sustainable development.

### 5.3 Promoting the coordinated development of community and family-based old-age care

The development of old-age care within communities and families is a critical strategy for addressing the challenges of population aging. This approach not only meets the growing demand for an improved quality of life for older adults but also stimulates the multidimensional development of the social economy. In May 2023, the Chinese government issued the “Opinions on Promoting the Construction of the Basic Old-Age Care Service System,” followed in October by the “Action Plan for Actively Developing Old-Age Meal Assistance Services.” These policy documents establish a clear framework for enhancing the old-age care service system and promoting community-based home care, thereby offering strategic directions to improve the well-being of older adults.

In addition to ensuring robust policy support, it is equally important to encourage market participation and the investment of social capital in old-age care (10). This involves developing infrastructure, creating care communities, building comprehensive care ecosystems, and expanding the industry to enhance both the material and spiritual well-being of older adults. As part of the basic old-age care service system and the promotion of home care services, a wide range of offerings should be provided (69). These include the development of products tailored to older adults, housekeeping services, and medical care. Additionally, homes for older adults who are disabled or semi-disabled should be renovated with age-friendly features, such as barrier-free facilities, to ensure safety and comfort in home-based care.

The rise of an aging society is also expected to foster new professions, such as bath assistants, health caregivers, and rehabilitation therapists (70), thereby opening up opportunities for younger generations to engage in the old-age care workforce. This development not only improves the quality of life for older adults but also creates new avenues for economic growth in related industries, driven by the increasing demand for high-quality care services.

Continued exploration and implementation of community-based and home-based old-age care are essential to ensure that the expanding old-age care economy aligns with the evolving needs of older adults. Such development must support sustainable growth in both economic and social spheres while addressing the challenges of an aging population.

### 5.4 Optimizing the precision of old-age social welfare through a collaborative governance mechanism

China is actively working to ensure that basic old-age care services are available to all older adults, addressing their diverse caregiving needs. The “Opinions on Strengthening the Work on the Elderly in the New Era” lays the foundation for a comprehensive old-age care service system. A national basic old-age care service catalog has been created, categorizing services into three main areas with 16 items, including material assistance, caregiving services, and social care services, to provide essential and inclusive support for older adults. This catalog aims to standardize care services, ensuring that service quality and standards evolve in tandem with the country’s economic and social development.

However, challenges remain in aligning these services with the actual needs of the older population, particularly in rural areas (71). The diverse and individualized demands of older adults often require more personalized care plans that extend beyond the broad categories outlined in the catalog (72). These plans must consider the varying health, financial, and social conditions of older adults to ensure that services are both relevant and effective.

Currently, the old-age care service system is largely dependent on government leadership and financial support. However, to enhance the overall system, it is crucial to strengthen the role of market mechanisms (73). By engaging private enterprises, China can encourage a more diverse range of services and improve both the availability and quality of care options. Innovations in service delivery—such as technology-enabled care solutions (74), private-sector partnerships (75), and nonprofit initiatives (10)—are instrumental in meeting the growing demand for old-age care services. Nonetheless, the practical implementation of these plans still requires further optimization to address the diverse needs of older adults. Enhancements in service delivery should focus on increasing efficiency, comprehensiveness, and quality, while ensuring that resources are allocated effectively to maximize impact.

Ultimately, achieving a sustainable and inclusive old-age care system will require ongoing collaboration among the government, market, and society. By strengthening social capital participation, enhancing the precision of social welfare services, and tailoring care to the specific needs of older adults, China can develop a more responsive and effective system that ensures dignity and support for all.

### 5.5 Strengthening the cultural development of old-age communities

China’s long-standing cultural tradition of caring for older adults, rooted in filial piety, has influenced the treatment of older generations within families and communities. This cultural norm (76), which expects children to support their aging parents, has been fundamental in meeting the basic material and emotional needs of older adults. However, as the population ages, it is increasingly important for both the government and society to adapt and strengthen support structures for older adults, particularly by enhancing their spiritual and cultural well-being.

The government has implemented a series of policies to support older adults by providing facilities and services that cater to their physical, emotional, and cultural needs. For instance, community centers offer a variety of cultural and educational activities designed to enrich the lives of older adults. These centers provide courses in calligraphy, painting, music, and other arts, enabling older adults to develop new hobbies, enhance their skills, and maintain an active lifestyle. Such activities promote mental well-being, instill a sense of achievement, and foster social integration (77). However, it is vital that services provided through these community cultural projects are regularly assessed and adapted to meet the evolving needs of older adults. Tailoring services to accommodate the diverse preferences and abilities of older adults—whether they favor educational, social, or physical activities—ensures that these programs deliver meaningful benefits and enhance their quality of life.

Encouraging older adults, their families, and social organizations to participate in community volunteer activities is another vital strategy. Such engagement not only fosters a sense of social identity and belonging but also helps older adults remain active and connected within their communities (78). Contributing to community initiatives provides them with a renewed sense of purpose and fulfillment, which is essential for maintaining mental health.

Physical health is as important as mental well-being, and providing appropriate fitness facilities and health guidance is essential to enable older adults to engage in activities that promote both physical and mental health (79). Activities such as Tai Chi, brisk walking, and dancing help improve flexibility, balance, and strength—factors that are crucial for preventing falls and enhancing overall mobility (80, 81). Additionally, these activities offer relaxation and stress relief, contributing to the mental health and vitality of older adults.

To create a truly supportive environment for older adults, the government should ensure that their material, spiritual, and cultural needs are fully met. This includes not only access to healthcare and financial support but also opportunities for cultural enrichment, personal growth, and social participation. Strengthening community cultural development and enhancing the cultural service supply within old-age social welfare programs are critical steps toward achieving these goals. Ultimately, improving the spiritual and cultural life of older adults is essential for recognizing their social value and fostering overall well-being.

### 5.6 Building a supply-driven old-age care service society

Based on the CGT analysis, the expected topic proportion for the seventh topic is significantly higher than that of the other six topics. This topic emphasizes the critical need for policies that optimize the supply of old-age care services to meet societal demand and promote overall social development. In the context of the old-age care system, a supply-driven approach involves adjusting and refining service provision in response to the growth, structural changes, and evolving needs of the aging population. Such an approach ensures that older adults have access to services that are suitable, convenient, and of high quality (82).

To create a diversified, multi-tiered, and supply-driven old-age care system, the government has introduced key policy documents such as the “Opinions on Strengthening the Construction of the Old-Age Care Service Workforce.” This initiative aims to develop a comprehensive system that cultivates a workforce both adequately sized and well-structured, with the skills needed to provide quality old-age care. Enhancing policy frameworks and strategic planning is essential to support ongoing service development (10). Additionally, private sector investment in old-age care services, along with innovations like smart care solutions and chain service models, can offer diverse care options (83). Establishing robust regulatory mechanisms—such as licensing and certification processes for old-age care institutions—is crucial to ensure service quality and safety. Overall, a supply-driven policy framework is vital for promoting social stability, while also driving sustainable economic development.

### 5.7 Emerging challenges in policy implementation

As aforementioned, effective policy implementation for equitable social welfare coverage requires both government reform and market cooperation. Economic growth should not come at the expense of social welfare progress (15). While the government has updated demographic policies and statutory retirement ages, the long-term sustainability of the social insurance fund remains uncertain. Without robust fiscal support and an adaptable monetary policy to fund infrastructure updates and public welfare facilities, it is questionable whether the private sector can meet the growing demand for old-age care in the coming decades. While these issues are discussed in existing literature, an emerging shift in global governance and its potential impact on the social welfare system has not been fully addressed.

Since 2019, trade tensions between the United States and China have posed a new challenge to World Trade Organization-based governance, driven by nationalism on both sides. While the trade war during the first Trump administration did not significantly affect the Chinese economy, assuming that the deteriorating trade relationship will not cause structural damage during the second Trump administration is risky. Although this forum does not address the economic hardships China may face amid shifting global governance, the potential drags for implementing a reformed social welfare system must not be overlooked. Below, we outline three pressing questions for future research on this topic:

First, if tariffs lead to higher unemployment, the number of contributors to basic pension insurance will decline. How would this affect the funding mechanism for the social insurance system? Second, if government revenues shrink due to reduced exports and weak domestic demand—already a concern in the past 2 years—the sustainability of the social security system will be threatened. In such cases, the government would likely become the last resort for funding, but it remains unclear whether monetary policy would permit a significant deficit to support the social security fund. Third, as China shifts from an export-driven economy to one driven by consumer spending, altered trade relationships could dampen both private sector and government tax revenues. This may reduce consumer spending, including among older adults, potentially harming the economy and undermining the social welfare funding mechanism. Ultimately, all proposed policy actions are contingent upon the funding mechanism, which faces increasing challenges under current international relations.

## 6 International relevance

While this paper focuses on a more equitable social welfare system for Chinese, its findings may be valuable for other nations facing similar demographic challenges. However, differences in political systems mean that the Chinese model cannot be directly adopted by other governments. The Chinese model is characterized by a top-level design—a government-led system that incorporates citizen participation in collaborative governance (84). In this framework, the government guarantees societal functions, societal forces act as intermediaries to advance government objectives, and citizens engage in the system to reap social welfare benefits. Essentially, the government secures its legitimacy by enhancing citizens’ quality of life through an implicit social contract, which is the institutional cornerstone of the Chinese path to modernization. Although democratically elected governments are similarly responsible for enhancing quality of life and social welfare, differences between market economies and centrally planned economies make some aspects of the Chinese experience less applicable. For example, a supply-side economic model is generally ineffective in developed economies (85), where old-age care services are seldom considered core public welfare and are predominantly delivered by decentralized private enterprises (86, 87).

Nonetheless, China’s strong emphasis on policy refinement remains universally relevant, and the current macro reality calls for convergence among different political systems in addressing demographic challenges. Traditional welfare models in both developed and developing economies are ill-equipped to address today’s socioeconomic challenges. For instance, the global rise in non-communicable diseases, combined with the high costs of treatment and follow-up management, not only burdens older adults (88, 89) but also contributes to social instability and fuels right-wing populism in many democracies (90). This challenge is further compromised by the post-COVID inflationary environment, which intensifies the financial hardship experienced by retirees. Moreover, an ongoing industrial revolution driven by artificial intelligence is triggering a labor transition, as traditional jobs become less cost-efficient compared to automation. In light of these trends, both central and liberal governance systems must adapt to new realities. Other liberal nations should urgently reassess their inherent weaknesses, implement legislative reforms to address unique social needs, and consider pivoting toward a universal basic social safety net. A centrally planned policy reform with private sector participation, similar to China’s top-level design model, could be efficient to address unprecedented socioeconomic transforms affecting older adults worldwide.

## 7 Limitations and future research

One criticism is that our topic model relies on abstracts rather than full texts. For example, a simulation study of LDA topic modeling indicate that differences between abstract and full-text data are more pronounced in smaller document collections, with up to 90% high-quality topics identified in full texts versus only 50% in abstracts (91). Although a well-written abstract should convey an article’s main theme, full texts might include additional topics. However, our study aims to capture the overall research direction, and thus abstracts are deemed sufficient for this purpose.

**Figure 5:**
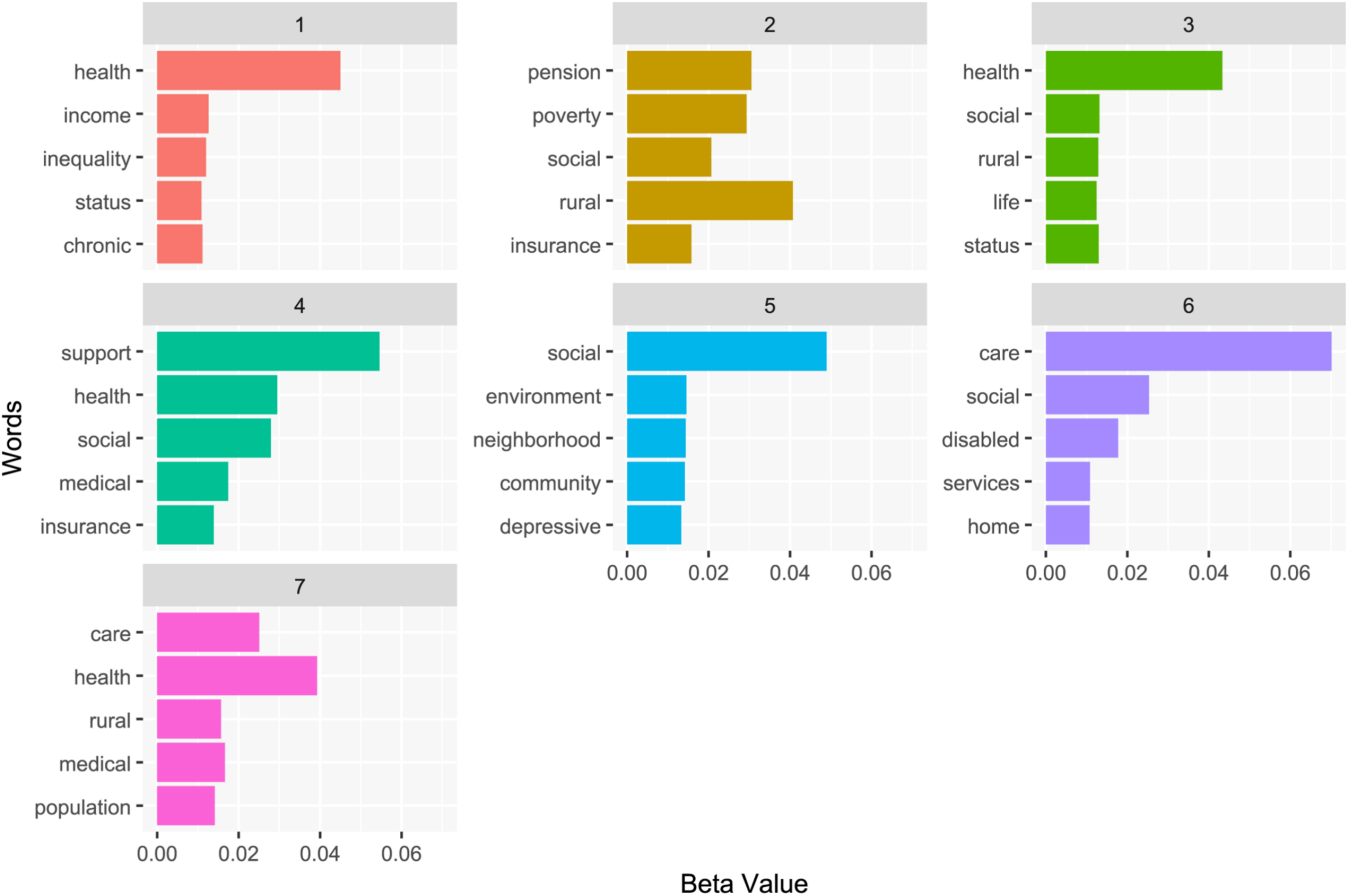
Top five terms for each CGT topic derived from the English literature on China’s old-age social welfare system.

The study only considered Chinese-language research articles, excluding significant English-language literature. To address this concern, we applied the same research framework (Figure 1) and conducted a separate analysis of English publications. We searched the Web of Science Core Collection using the Boolean expression (“social security” OR “social insurance” OR “social welfare”) AND (“old age” OR “older adults” OR “older people” OR “elderly”) AND “China,” yielding 647 documents. Of these, 256 documents were included in the analysis (DOI: 10.6084/m9.figshare.28029059.v4). Figure 5 displays the top five keywords per topic based on our modified CGT approach, revealing both similarities and differences compared to the main results. Specifically, topics two, four, five, and six address the social welfare system for rural old-age care (aligned with topic two in Table 1), old-age care services and policies (aligned with topic five in Table 1), community-and family-based care (aligned with topic four in Table 1), and the old-age care needs of people with disabilities (aligned with topic five in Table 1), respectively. These findings indicate robust coverage of research from both Chinese and English publications. However, topics one, three, and seven, which focus on old-age care support for chronic health conditions, are less detailed in our analysis. This discrepancy likely stems from our emphasis on the macro aspects of the social welfare system, which may have overlooked more granular issues. Future studies focusing on specific areas, such as basic medical insurance, could yield more targeted insights.

While this study represents the first attempt to apply CGT to analyze China’s social welfare system, it faces unavoidable drawbacks inherent to this novel method. Our reliance on LDA topic modeling introduces a degree of opacity, as the process of selecting algorithm parameters lacks standardized guidelines, making it somewhat of a “black box” for non-computer scientists. Several questions remain unanswered: How should stop words and stem words be selected during pre-processing, given their potential impact on word frequencies and analysis outcomes? Is there a reliable criterion for determining the “best” number of topics when adjusting parameters? Could a modified Dirichlet distribution offer improved performance in identifying latent topics (92)? Moreover, should LDA be compared with other algorithms (34), and if so, what criteria should determine the superior method? These unresolved issues are critical to the validity of our findings, and we urge readers to interpret results generated by the current algorithm with caution.

Current natural language processing has achieved unprecedented efficiency. For instance, it takes less than 15 s to analyze 256 documents (DOI: 10.6084/m9.figshare.28029059.v4). In the future, the rapid advancement of pre-trained large language models (LLMs) holds the potential to further revolutionize natural language processing and enhance reproducibility in this field. We propose that computer scientists and statisticians collaborate to develop LLMs specifically tailored for topic modeling. Ideally, these models would allow researchers to clearly define research questions and automate tasks such as document retrieval (e.g., database searches and data import), pre-processing (e.g., optical character recognition, stop word suggestions), topic modeling (e.g., automatic selection of the optimal number of topics), and pattern refinement. Leveraging LLMs could mitigate human bias, eliminate the need for programming expertise, and significantly reduce the time and cost associated with qualitative research. Thus, LLMs may represent a transformative breakthrough for social science research, paving the way for unprecedented efficiency and rigorous reproducibility of results.

## 8 Conclusion

This study systematically analyzes China’s existing literature on the old-age social welfare system, identifying seven thematic topics. Given the predominance of research on rural old-age care, it indicates that significant disparities persist between economically disadvantaged agricultural regions and more globalized urban economies. Meanwhile, much research focuses on supply-driven old-age care services, reflecting China’s top-level design governance model, although the effectiveness of these supply-side services for older adults remains tested. Utilizing a novel CGT framework, the study highlights six targeted areas from which policy priorities can be established to build a more equitable and sustainable social welfare system for China’s growing older population.

An added value of this study is its detailed demonstration of a computer-assisted approach that information professionals, including social scientists, can adopt. Beyond its application in systematic reviews, this method is especially valuable for mining large, unstructured datasets—such as newspapers, interviews, and social media posts—in social science research. By harnessing the power of artificial intelligence, researchers can reduce the need to read full texts and instead focus on identifying latent themes, all while minimizing bias. It is anticipated that both researchers and policymakers will increasingly leverage advanced artificial intelligence technologies to tackle diverse challenges in fostering a fairer society.

## Data Availability

Publicly available datasets were analyzed in this study. This data can be found at: a complete list of the included documents is available on Figshare (DOI: 10.6084/m9.figshare.28029059.v2).
